# Supplementary material for: The interactome of intact mitochondria by cross-linking mass spectrometry provides evidence for coexisting respiratory supercomplexes
Source: Mol Cell Proteomics. 2017 Dec 8;17(2):216–32. doi: 10.1074/mcp.RA117.000470 (PMC5795388; doi:10.1074/mcp.RA117.000470)
Supplement: Supplemental Data [file supp_RA117.000470_133922_0_supp_23978_fzffwf.zip › spectra_annotation/mito_DR_spectra_annotation/84-1-4-1-4-1.pdf]

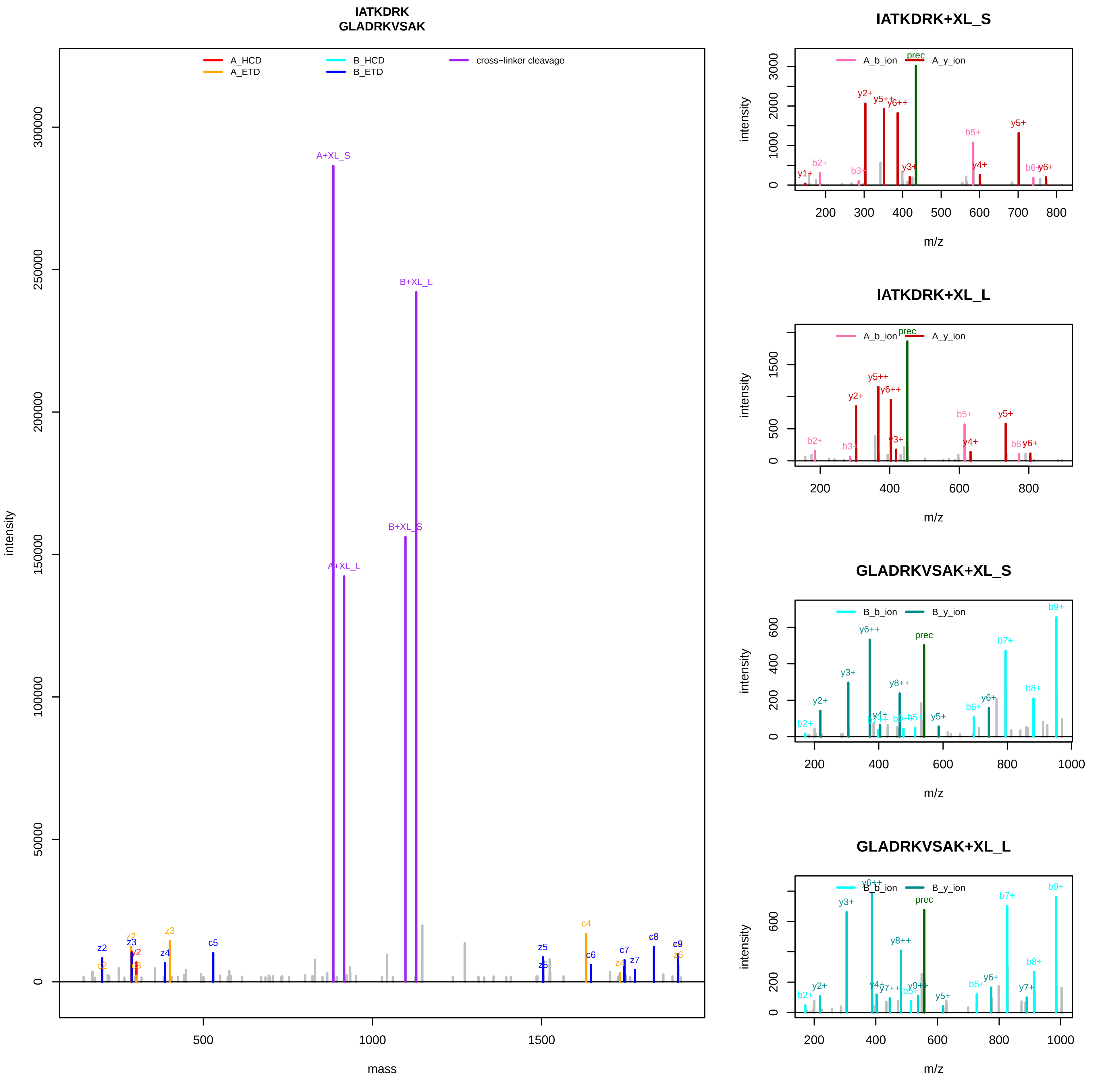
IATKDRK
GLADRKVSAK
A_HCD
A_ETD
B_HCD
B_ETD
cross−linker cleavage
A+XL_S
B+XL_L
B+XL_S
A+XL_L
intensity
mass
0
50000
100000
150000
200000
250000
300000
500
1000
1500
z2
z3
y2
z4
z3
c5
z5
c4
c6
c7
z7
c8
c9
IATKDRK+XL_S
IATKDRK+XL_L
GLADRKVSAK+XL_S
GLADRKVSAK+XL_L
A_b_ion
A_y_ion
B_b_ion
B_y_ion
prec
m/z
y1+
b2+
b3+
y2+
y5++
y6++
y3+
b5+
y4+
y5+
b6+
y6+
y3+
y8++
b7+
b8+
b9+
y7+
